# PROTEIN SUPPLEMENTATION DOES NOT RESCUE MUSCLE MASS AND FUNCTION–HEAVY RESISTANCE TRAINING IS REQUIRED

**DOI:** 10.1093/geroni/igz038.332

**Published:** 2019-11-08

**Authors:** Lars Holm, Rasmus Bechshoeft, Soren Reitelseder, Kenneth Mertz, Jacob Bulow, Grith Hojfeldt

**Affiliations:** 1 University of Birmingham, Birmingham, UK, United Kingdom; 2 Institute of Sports Medicine, Bispebjerg Hospital, Copenhagen, Denmark, Denmark

## Abstract

The requirement of an enhanced dietary protein intake to counteract the age-related loss of muscle mass is still debated. Further, the dinner meal generally contains the majority of protein and energy and since, the muscle of older adults responds less to protein intake than that of younger adults it is hypothesized that older adults would benefit from taking more protein in at other meals. The aim of this study was to investigate whether the provision of protein supplements for breakfast and lunch meals over the course of a year would make healthy, older, home-dwelling adults (N=136) take in more protein and whether that then would affect their muscle mass (primary outcome) and a number of metabolic health parameters, muscle strength parameters and functional capabilities. More than 77% ingested more than 75% of the provided supplements, irrespective of supplementation type (isocaloric carbohydrate; collagen hydrolysate low quality protein: whey hydrolysate high quality protein). Providing supplementation for a year among older adults makes them comply very well. However, provision of extra protein has no impact on the muscle mass or strength or on the functional parameters. Further, we studied the impact of adding resistance training on top of WHEY protein supplementation and found that heavy more than light-load resistance training affects fat-free mass and maximal-voluntary contraction. Daily protein intake can be enhanced by supplementation but do not impact muscle mass and function over the course of a year, where heavy resistance training on top benefits, but to a lesser than expected degree.

